# Successful Closure of Persistent Urinary Fistula After High‐Grade Renal Trauma Using N‐Butyl‐2‐Cyanoacrylate: A Case Report

**DOI:** 10.1002/iju5.70239

**Published:** 2026-07-27

**Authors:** Yui Yamauchi, Masataka Kawamura, Yuji Koretsune, Kentaro Takezawa, Taigo Kato, Koji Hatano, Yoichi Kakuta, Atsunari Kawashima, Yusuke Ono, Norio Nonomura

**Affiliations:** ^1^ Department of Urology The University of Osaka Graduate School of Medicine Osaka Japan; ^2^ Department of Diagnostic and Interventional Radiology The University of Osaka Graduate School of Medicine Osaka Japan

## Abstract

**Introduction:**

Persistent urinary extravasation after high‐grade renal trauma is uncommon but may require intervention. Minimally invasive, kidney‐preserving options are particularly desirable in young patients.

**Case Presentation:**

An 18‐year‐old man sustained blunt trauma in a motorcycle accident. Contrast‐enhanced computed tomography revealed renal injury with urinary extravasation. Emergency transcatheter arterial embolization was performed for hemorrhage control. Despite subsequent ureteral stenting and percutaneous drainage, urinary leakage persisted. Fistulography demonstrated a small fistulous tract between the urinoma cavity and the lower calyx. Selective embolization using N‐butyl‐2‐cyanoacrylate (NBCA) was performed, resulting in immediate resolution without complications.

**Conclusion:**

Selective NBCA embolization is a safe and minimally invasive treatment option for persistent urinary fistula after renal trauma.

## Keynote Message

Persistent urinary leakage after renal trauma does not necessarily require surgical reconstruction or nephrectomy; selective NBCA embolization can achieve renal preservation when the fistulous tract is small and well localized and should be considered before surgical intervention.

## 1 Introduction

Non‐operative management has become the standard treatment for most renal trauma, even in high‐grade injuries [1]. Urinary extravasation occurs in approximately half of patients with high‐grade renal trauma [2]; however, most cases can be managed successfully, with ureteral stenting and drainage.

Persistent urinary leakage despite conservative management is relatively uncommon, but when it occurs, management remains challenging [3]. Surgical repair or nephrectomy may be required in refractory cases, although these procedures are invasive and may compromise renal function, particularly in young patients.

NBCA is a liquid embolic agent widely used in both vascular and non‐vascular interventional procedures [4]. Recently, its use has been reported in the management of urinary fistulas, particularly after partial nephrectomy or urologic surgery [5]. However, reports describing its application in traumatic urinary fistulas remain limited.

Here, we present a case of persistent urinary leakage after high‐grade renal trauma that was successfully treated with selective NBCA embolization.

## 2 Case Presentation

**FIGURE 1:**
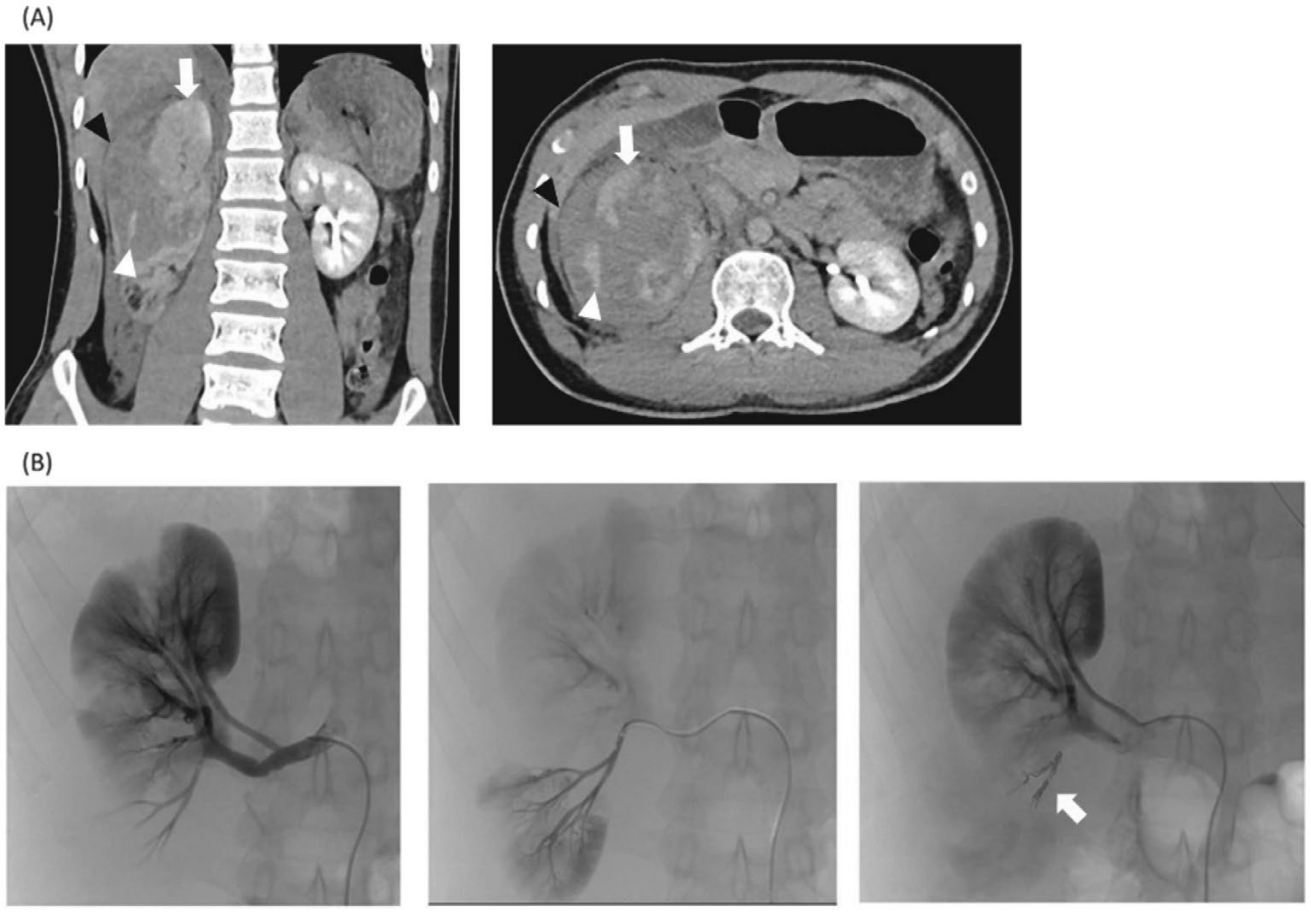
(A) Contrast‐enhanced computed tomography on the day of injury demonstrating a right renal parenchymal hematoma with urinary extravasation extending into the retroperitoneal space. White arrow: Renal parenchyma, Black arrow head: Hematoma, White arrow head: Urinary extravasation. (B) Angiographic findings during TAE demonstrating separation of the lower pole from the main renal parenchyma; coil embolization of the lower pole branch (arrow) was performed with adjunctive use of a small amount of gelatin sponge slurry.

An 18‐year‐old man was transported to our hospital after a motorcycle collision with a passenger car. On arrival, his blood pressure was 97/50 mmHg and pulse rate was 120 beats per minute. Contrast‐enhanced computed tomography demonstrated a large right renal parenchymal hematoma with urinary extravasation extending into the retroperitoneal space (Figure 1A). Separation of the lower pole from the main renal parenchyma was observed, and the injury was classified as American Association for the Surgery of Trauma (AAST) grade IV renal injury. Although right renal angiography showed no definite active contrast extravasation, emergency TAE was performed on the day of injury to decrease urine production from the separated lower pole (Figure 1B). Angiography revealed disruption of the lower pole branch of the renal artery, and coil embolization was performed with adjunctive use of a small amount of gelatin sponge slurry.

**FIGURE 2:**
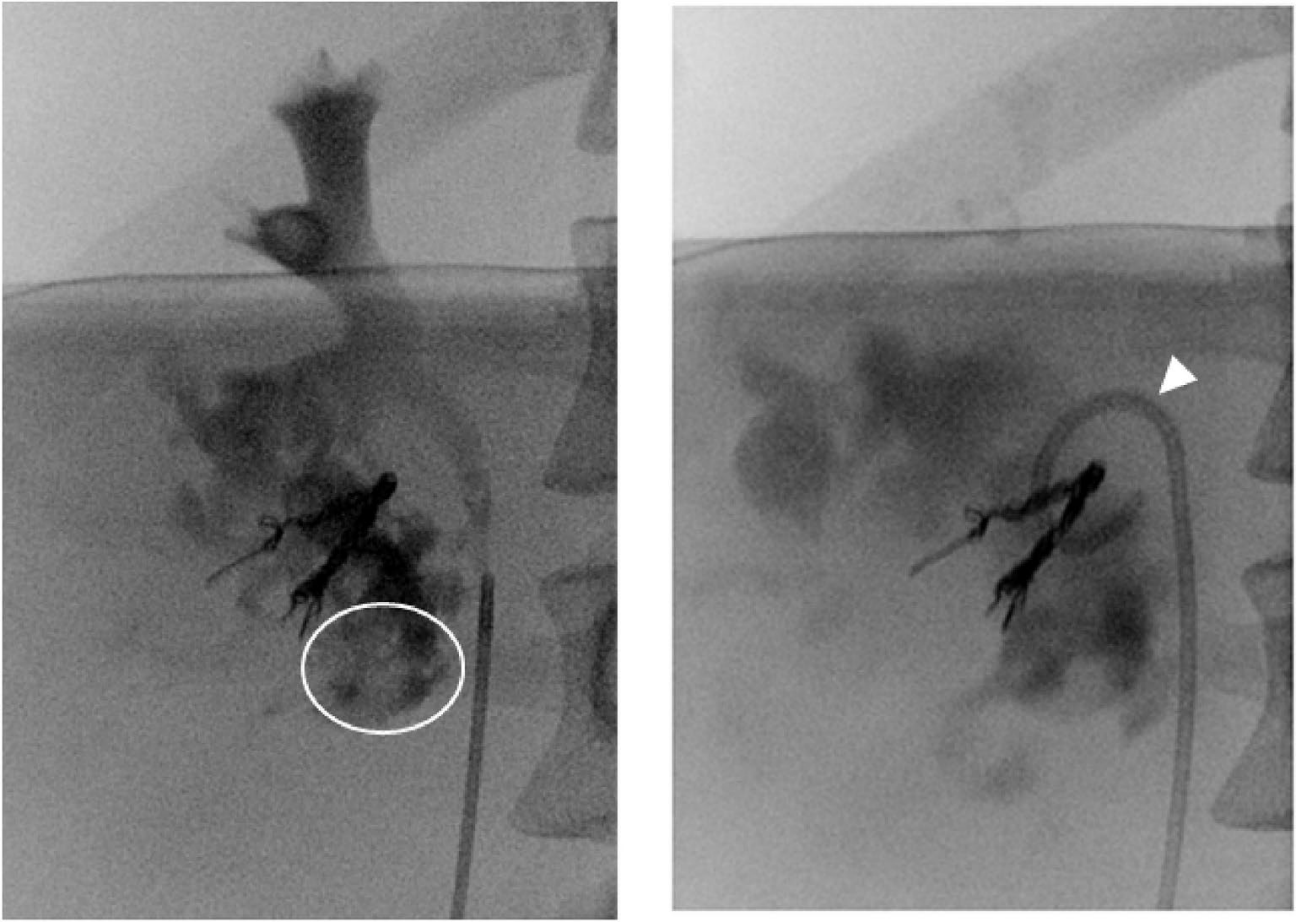
Retrograde pyelography performed on day 9 after injury demonstrating urinary leakage from the mid calyx tracking toward the lower pole (circle); a double‐J ureteral stent (arrow head) was subsequently placed.

After hemodynamic stabilization, further evaluation was performed. Retrograde pyelography on day 9 demonstrated urinary leakage from the mid calyx toward the lower pole (Figure 2), and a double‐J ureteral stent was placed. However, leakage persisted, and percutaneous drainage of the urinoma was performed on day 26 using an 8.5‐Fr pigtail catheter. Drain output remained 100–300 mL/day.

**FIGURE 3:**
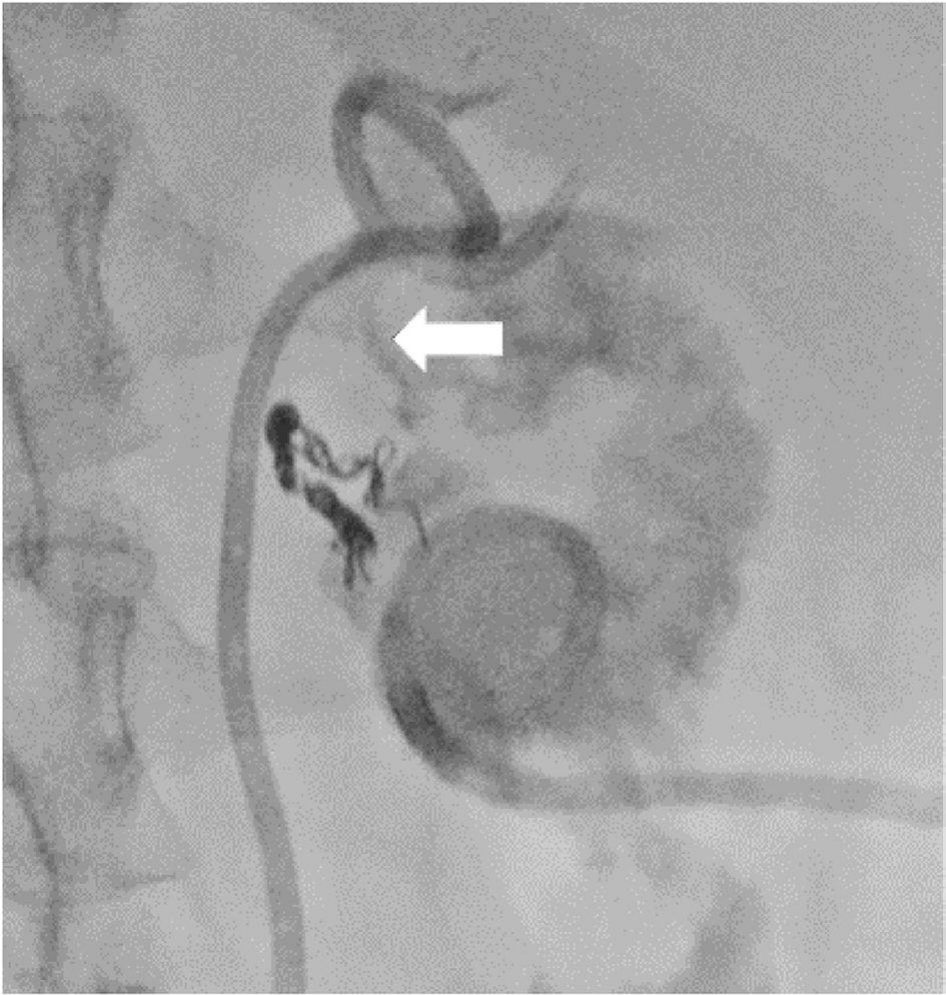
Fistulography performed on day 45 after injury demonstrating communication between the urinoma cavity and the lower calyx, confirming the presence of a urinary fistula. The arrow indicates the fistulous tract.

Fistulography on day 45 demonstrated communication between the urinoma and the lower calyx, confirming a urinary fistula (Figure 3). Conservative management was continued, but leakage persisted for more than 60 days. Repeat imaging showed a short, narrow fistulous tract with a longitudinal length of approximately 5 mm. Given its small and well‐localized nature, embolization using NBCA was considered feasible.

**FIGURE 4:**
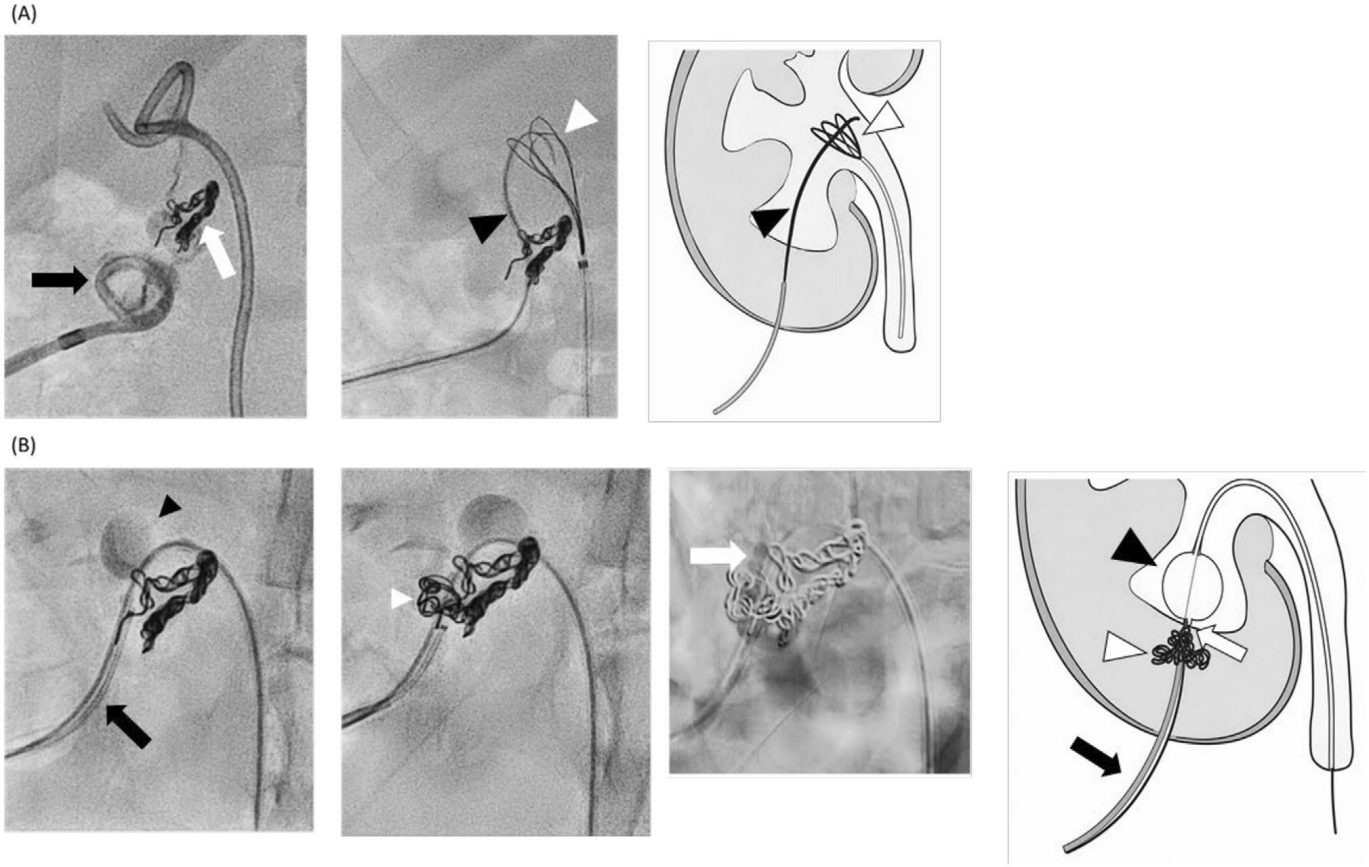
Selective embolization of the urinary fistula. (A) A guidewire (black arrowhead) was advanced through the fistulous tract communicating with the lower calyx and captured with a snare (white arrowhead) introduced from the urinary tract, creating through‐and‐through access. The black arrow indicates the 8.5‐Fr drainage catheter placed in the urinoma cavity. The coil mass (white arrow) visible near the lower pole was placed during transcatheter arterial embolization on the day of injury. (B) A balloon catheter (black arrow head) was positioned advanced over the guidewire on the calyceal side while embolization coils (white arrow head) through a microcatheter (black arrow) advanced over the guidewire were deployed on the extrarenal side to sandwich the fistulous tract and prevent migration of NBCA. Subsequently, 0.5 mL of 66% NBCA was injected into the fistulous tract to achieve complete closure. The white arrow indicates NBCA injected into the fistulous tract. For clarity, both schematic illustrations in (A) and (B) depict a normal kidney anatomy; the lower calyx separated as a result of the traumatic injury is not shown.

On day 61, fistula embolization was performed under fluoroscopic guidance. The fistulous tract communicating with the lower calyx was cannulated with a 0.035‐in. Radifocus guidewire (Terumo, Tokyo, Japan) (Figure 4A), which was captured using a 6‐Fr En Snare (Merit Medical, South Jordan, UT, USA) introduced from the urinary tract to establish through‐and‐through access. A balloon catheter (Selecon MP Catheter II, Terumo, Tokyo, Japan) was positioned on the calyceal side to prevent migration into the collecting system (Figure 4B). Tornado embolization coils (Cook Medical, Bloomington, IN, USA) through a microcatheter (Master HF/KIT, Asahi Intec, Aichi, Japan) advanced over the guidewire were deployed on the extrarenal side to stabilize the tract and provide a scaffold. Subsequently, 0.5 mL of an NBCA–Lipiodol mixture (2:1) was injected into the fistulous tract through the same microcatheter. Additional coils and a second NBCA injection were required due to residual leakage. Both the balloon catheter and the microcatheter were advanced over the guidewire and stabilized by the through‐and‐through configuration. Final imaging confirmed complete occlusion.

Drain output rapidly decreased and ceased completely. Follow‐up fistulography 2 weeks later confirmed closure. The patient was discharged on day 78. No recurrence was observed, and the ureteral stent was removed 3 months later with preserved renal function.

## 3 Discussion

In the present case, persistent urinary leakage after grade IV renal trauma persisted for more than 60 days despite ureteral stenting and percutaneous drainage. Selective NBCA embolization with adjunctive balloon protection and coil packing successfully achieved complete fistula closure while preserving renal function. NBCA is a liquid embolic agent that rapidly polymerizes upon contact with ionic fluids such as blood or urine [6]. Previous reports have described the use of NBCA embolization mainly for urinary fistulas following partial nephrectomy or other urologic procedures [7]. In contrast, reports describing its application to traumatic urinary fistulas remain limited. Although most urinary extravasations after high‐grade renal trauma resolve with conservative management, persistent urinary leakage may lead to prolonged drainage, repeated interventions, infectious complications, and long‐term ureteral stent placement [8, 9]. In refractory cases, surgical repair or nephrectomy may be required [10]. However, these procedures are invasive and may compromise renal function, particularly in young patients. Compared with surgical treatment, selective fistula embolization offers several advantages. It is minimally invasive, preserves renal parenchyma, and avoids the morbidity associated with reconstructive surgery or nephrectomy. Another important consideration is the timing of intervention. In this patient, urinary leakage persisted for more than 60 days despite ureteral stenting and percutaneous drainage, suggesting fistula maturation and a low likelihood of spontaneous closure. Delayed embolization was therefore considered appropriate. Technical factors may also have contributed to successful treatment. The fistulous tract was short (approximately 5 mm in length), allowing precise embolization [11]. In addition, balloon protection on the calyceal side and coil packing on the extrarenal side may have prevented migration into the collecting system and improved retention of NBCA. The main limitation of this report is that it describes a single case. Further studies are needed to clarify the indications, technical considerations, and long‐term safety of NBCA embolization for traumatic urinary fistulas.

## 4 Conclusion

Selective embolization using NBCA can be an effective and minimally invasive treatment option for persistent urinary fistula following high‐grade renal trauma. When the fistulous tract is narrow and well localized, this approach may allow renal preservation and avoid the need for surgical intervention.

## Ethics Statement

The authors nothing to report.

## Consent

Written informed consent was obtained from the patients for the publication of this case report and the accompanying images.

## Conflicts of Interest

Kentaro Takezawa is an Editorial Board member of the *International Journal of Urology* and a co‐author of this article. To minimize bias, he was excluded from all editorial decision‐making related to the acceptance of this article for publication. The other authors declare no conflicts of interest.

## Data Availability

Data sharing is not applicable to this article as no datasets were generated or analyzed.
